# Detection of antimicrobial producing *Staphylococcus* from migratory birds: Potential role in nasotracheal microbiota modulation

**DOI:** 10.3389/fmicb.2023.1144975

**Published:** 2023-04-11

**Authors:** Rosa Fernández-Fernández, Idris Nasir Abdullahi, Carmen González-Azcona, Adriana Ulloa, Agustí Martínez, Sara García-Vela, Ursula Höfle, Myriam Zarazaga, Carmen Lozano, Carmen Torres

**Affiliations:** ^1^Area of Biochemistry and Molecular Biology, One Health-UR Research Group, University of La Rioja, Logroño, Spain; ^2^Department of Food Science, University of Laval, Québec City, QC, Canada; ^3^SaBio (Health and Biotechnology) Research Group, Game and Wildlife Research Institute, Spanish National Research Council/University of Castilla–La Mancha, Ciudad Real, Spain

**Keywords:** staphylococci, coagulase-negative staphylococci, bacteriocins, antimicrobial activities, storks, nasal microbiota

## Abstract

A collection of 259 staphylococci of 13 different species [212 coagulase-negative (CoNS) and 47 coagulase-positive (CoPS)] recovered from nasotracheal samples of 87 healthy nestling white storks was tested by the *spot-on-lawn* method for antimicrobial-activity (AA) against 14 indicator bacteria. Moreover, extracts of AP isolates were obtained [cell-free-supernatants (CFS) both crude and concentrated and butanol extracts] and tested against the 14 indicator bacteria. The microbiota modulation capacity of AP isolates was tested considering: (a) intra-sample AA, against all Gram-positive bacteria recovered in the same stork nasotracheal sample; (b) inter-sample AA against a selection of representative Gram-positive bacteria of the nasotracheal microbiota of all the storks (30 isolates of 29 different species and nine genera). In addition, enzymatic susceptibility test was carried out in selected AP isolates and bacteriocin encoding genes was studied by PCR/sequencing. In this respect, nine isolates (3.5%; seven CoNS and two CoPS) showed AA against at least one indicator bacteria and were considered antimicrobial-producing (AP) isolates. The AP isolates showed AA only for Gram-positive bacteria. Three of these AP isolates (*S. hominis* X3764, *S. sciuri* X4000, and *S. chromogenes* X4620) revealed AA on all extract conditions; other four AP isolates only showed activity in extracts after concentration; the remaining two AP isolates did not show AA in any of extract conditions. As for the microbiota modulation evaluation, three of the nine AP-isolates revealed intra-sample AA. It is to highlight the potent inter-sample AA of the X3764 isolate inhibiting 73% of the 29 representative Gram-positive species of the nasotracheal stork microbiota population. On the other hand, enzymatic analysis carried out in the two highest AP isolates (X3764 and X4000) verified the proteinaceous nature of the antimicrobial compound and PCR analysis revealed the presence of lantibiotic-like encoding genes in the nine AP isolates. In conclusion, these results show that nasotracheal staphylococci of healthy storks, and especially CoNS, produce antimicrobial substances that could be important in the modulations of their nasal microbiota.

## 1. Introduction

Bacteria thrive in complex niches establishing inter-species and intra-environment relationships, including the ability to acquire and transfer several adaptation mechanisms (Krismer et al., 2017; Heilbronner et al., 2021). The nasal cavity is directly connected to the external media and in close contact with a wide diversity of microorganisms that can be acquired through inhalation (Biswas et al., 2015). Moreover, the microenvironment of the nasal cavity varies depending on the anatomical location.

The anterior nares (nostrils) are the most difficult area for the survival of microbes due to their acidic environment with high salinity (Geurkink, 1983). Therefore, microbes that live in the nasal cavity are subjected to a variety of stress conditions and they must counteract to survive and persist (Krismer et al., 2014). In this respect, species competition in the nasal cavity can be mediated by direct or indirect mechanisms such as the acquisition of nutrients, the production of antimicrobial substances and the activation of specific host defense mechanisms (Krismer et al., 2017).

Human nasal microbiota is mainly composed of *Staphylococcus, Cutibacterium, Corynebacterium*, and *Moraxella* (Zhou et al., 2014), and bacteria from other genera are less frequently found (Liu et al., 2015). Focusing on the *Staphylococcus* genus, human nasal isolates have been frequently described as producers of antimicrobial substances against bacterial competitors. For instance, *S. epidermidis* or *S. lugdunensis*, among others favorably ousted *S. aureus* (Janek et al., 2016; Zipperer et al., 2016). However, there could be limitations in the detection and/or production of antimicrobial activity due to some producing isolates require specific environmental stress conditions commonly present in the human nose, such as hydrogen peroxide release and iron limitation (Janek et al., 2016).

The nasal microbiota of animals has also been analysed revealing composition differences. For example, companion and farm animals or rodents have higher abundances of Proteobacteria as compared to humans (Weese et al., 2014; Chaves-Moreno et al., 2015; Misic et al., 2015), and some *S. aureus* lineages, including livestock-associated MRSA, are increasingly found in the noses of livestock (Bal et al., 2016). However, staphylococci and especially coagulase-negative staphylococci (CoNS) from wildlife remain largely understudied. In this respect, some studies carried out in Spain and Portugal revealed that wild animals (birds and mammals) are frequently colonized by CoNS and *S. sciuri* being one of the predominant species among this group of microorganisms (Sousa et al., 2016; Mama et al., 2019; Ruiz-Ripa et al., 2020).

Birds have been postulated as sentinels, reservoirs, and potential disseminators of antimicrobial resistance due to their interaction with the human interface, diverse ecological niches, and capacity to travel for long distances (Bonnedahl and Järhult, 2014). Consequently, the nasotracheal bacterial communities of white storks have recently been studied by our research group (Abdullahi et al., 2023). Moreover, storks can also be a source of antimicrobial substances thanks to the adaptation strategies of the isolates present in their bacterial communities. Here, the present study aims to detect and partially characterize the production profile of antimicrobial substances in *Staphylococcus* isolates from nasotracheal samples of nestling white storks obtained from a previous study (Abdullahi et al., 2023), and to evaluate their capacity as modulators of the nasotracheal microbiota of these animals.

## 2. Materials and methods

### 2.1. Staphylococcal isolates used for the detection of antimicrobial activity (AA)

A total of 259 Staphylococcal isolates of stork origin were included in this study and they were tested for the production of antimicrobial activity (AA). These isolates were of 13 different species (number of isolates): *S. aureus* (46), *S. sciuri* (138), *S. epidermidis* (16), *S. lentus* (14), S. *chromogenes* (11), *S. xylosus* (8), *S. hominis* (7), *S. simulans* (7), *S. saprophyticus* (6), *S. haemolyticus* (3), *S. hyicus* (1), *S. capitis* (1), and *S. arlettae* (1).

These 259 Staphylococcal isolates were obtained from 136 samples (84 tracheal and 52 nasal) of 87 nestling white storks in a previous study (Abdullahi et al., 2023). The animals included belonged to four different colonies of storks located in South-central Spain. For bacterial isolation, the nasal or tracheal samples were pre-enriched in brain heart infusion broth supplemented with 6.5% NaCl and after overnight incubation, four culture media were used for bacteria recovery [blood agar, mannitol salt agar, oxacillin screening agar base supplemented with oxacillin (ORSAB medium), and CHROMagar™ LIN]. Finally, up to 12 different colonies were randomly selected per sample and identified by matrix-assisted laser desorption/ionization time-of-flight mass spectrometry (MALDI-TOF-MS; Bruker Daltonics, Bremen, Germany). Thus, 259 distinct staphylococci were included in the present study (one isolate of each *Staphylococcal* species per animal), which corresponded to 2–5 staphylococci/animal.

### 2.2. Isolates used as indicator bacteria in the screening for detection of antimicrobial-producing (AP) staphylococci

Fourteen Gram-positive (G+) isolates of different genera and species were used as indicator bacteria to evaluate the AA of the collection of 259 *Staphylococcal* isolates of storks. The list of these 14 indicator bacteria is shown in Supplementary Table 1 and includes relevant pathogenic, zoonotic, and multidrug-resistant (MDR) bacteria methicillin-resistant *S. aureus* (MRSA) (C1570), methicillin-susceptible *S. aureus* (MSSA) (ATCC29213), methicillin-resistant *Staphylococcus pseudintermedius* (MRSP) (C2381), methicillin-susceptible *S. pseudintermedius* (MSSP) (C3468), *Staphylococcus lugdunensis* (C10107), *S. epidermidis* (C2663), *S. sciuri* (C9780), *Staphylococcus delphini* (C9459), *Enterococcus cecorum* (X3809), *Enterococcus faecalis* (ATCC29212), *Enterococcus faecium* (C2321), *Micrococcus luteus* (CECT241), *Listeria monocytogenes* (CECT4032), and *Streptococcus suis* (X2060). Two additional Gram-negative (G–) indicator bacteria were tested in the AP staphylococci detected: *Escherichia coli* (ATCC25922) and *P. aerugino*sa (PAO1).

### 2.3. Detection of antimicrobial activity (AA)

Four methods were used to determine antimicrobial activity:

1. *Spot-on-lawn* method: Indicator isolates were resuspended in Brain Heart Infusion broth (BHI; Condalab, Madrid, Spain) up to 0.5 MacFarland and 10 μL of the cultures were added to five-milliliter aliquots of Tryptic Soy Broth supplemented with 0.3% yeast extract (TSB; Condalab, Madrid, Spain) and 0.7% agar tempered at 45°C. Then, the mixture was seeded onto plates containing Tryptic Soy agar plus 0.3% yeast extract (TSA; Condalab, Madrid, Spain), and the putative antimicrobial-producing (AP) isolates were spotted on the surface and plates were incubated overnight at 37°C. When *Streptococcus suis* was the indicator bacteria, Columbia agar with 5% sheep blood (bioMérieux SA, France) was used instead of TSA. Growth inhibition was detected by a clearing zone with no bacterial growth around the AP isolate.
2. Crude cell-free supernatant (CFS): Antimicrobial-producing isolates were grown in 10 mL of BHI medium for 24 h at 37°C, centrifuged (4.500 rpm, 10 min) and sterilized by boiling or filtration through a low-protein binding 0.45 μm Millipore filter.
3. Concentrated CFS: The resulting crude CFS was concentrated by speed vacuum and resuspended in dimethyl sulfoxide (DMSO).
4. Butanol extraction: 1-butanol was added to a fresh overnight BHI broth culture at a ratio of 1:2 and shacked for 1 h at 37°C. After phases differentiation, samples were centrifuged at 4.500 rpm for 15 min. The organic phase was tested for antimicrobial activity.

For methods 2–4, 50 μL of the *Staphylococcal* extracts were filled on wells done on TSA agar plates previously inoculated with the indicator bacteria, and the plates were incubated for 24 h at 37°C. The AA was assessed by the analysis of the inhibition zones around the wells. Positive (a previously described AP-isolate) and negative (BHI medium, DMSO, butanol) controls under all conditions were included in the assays.

### 2.4. Analysis of microbiota modulation

In the Staphylococcal isolates that showed antimicrobial activity by the *spot-on-lawn* method (AP isolates), their antimicrobial activity was also analysed against G^+^ bacteria obtained from the storks’ samples (used in this case as indicator bacteria), using the same procedure. Two different approaches were used:

1. Intra-sample activity. The activity of the AP isolates against all the G+ bacteria recovered in the same stork nasotracheal sample of the selected AP isolate was analyzed.
2. Inter-sample activity. From the whole collection of bacteria obtained from the nasotracheal samples of the 87 storks included in this study (Abdullahi et al., 2023), one isolate of each G^+^ species was selected (avoiding the selection as representants of the isolates recovered in the same samples in which AP bacteria were detected). Following these criteria, a collection of 30 isolates of 29 different G^+^ species and nine genera was used as indicator bacteria (representative of the inter-sample community) (Supplementary Table 2).

### 2.5. Characterization of the antimicrobial compounds in AP staphylococci

1. Susceptibility to proteolytic enzymes. The following enzymes were assayed (treatment conditions): trypsin (pH = 7.6; 25°C), α-chymotrypsin (pH = 7.8; 25°C), proteinase-K (pH = 7.5; 37°C), papain (pH = 6.2; 25°C) and protease (pH = 7.5; 37°C) (Sigma). Boiled CFS of two selected AP isolates (*S. sciuri* X4000 and *S. hominis* X3764) were prepared as described above. After adjusting to optimal pH, aliquots were independently incubated for 1 h with 1 g/L of each enzyme. After treatment, the enzymes were inactivated by boiling and antimicrobial activity was assayed (Navarro et al., 2000). Hemoglobin was used as negative control in all assays under all conditions.
2. Bacteriocin gene detection. The presence of 22 bacteriocin structural genes was tested by PCR and sequencing in all the AP-isolates detected in this study (*aurA, aucA, epiA, sacaA/sacbA, gdmA, bacSp222, nsj, hyiA, hycS, bacCH91, bsaA2, acIA, ale-1, lss, nukA, nkqA, eciA, pepA, elxA, elkA, ecdA, orf4*), as well as three bacteriocin gene families described elsewhere (BS, GEST, and NUK) (Fernández-Fernández et al., 2022).

### 2.6. Antibiotic resistance phenotype of AP isolates

The susceptibility to 13 antibiotics was evaluated in the AP isolates by the disk diffusion method and they were interpreted using the European Committee on Antimicrobial Susceptibility Testing criteria (EUCAST, 2022). The antibiotics tested were as follows: penicillin, cefoxitin, oxacillin, erythromycin, clindamycin, gentamicin, tobramycin, tetracycline, ciprofloxacin, chloramphenicol, linezolid, trimethoprim–sulfamethoxazole, and mupirocin.

## 3. Results

### 3.1. Antimicrobial activity of the collection of staphylococci isolates of storks against Gram-positive bacteria

**FIGURE 1:**
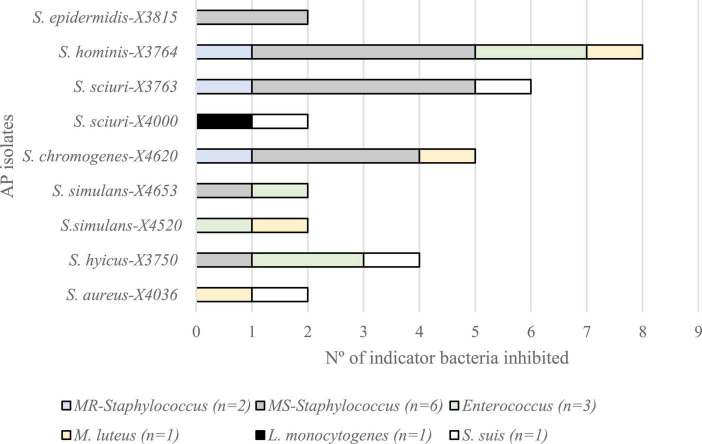
Antimicrobial activity of the nine antimicrobial producer (AP) isolates against 14 Gram-positive indicator bacteria grouped in six categories (*n*, number of isolates of each category), by the *spot-on-lawn* method.

Antimicrobial activity was detected by the *spot-on-lawn* method in 9 of the 259 staphylococci (3.5%) of stork origin tested, and they were considered as AP-isolates: *S. aureus* X4036, *S. hyicus* X3750, *S. sciuri* X3763 and X4000, *S. epidermidis* X3815, *S. chromogenes* X4620, *S. hominis* X3764, and *S. simulans* X4520 and X4653 (Table 1). These AP isolates were obtained from seven different storks and revealed AA against at least one of the 14 G^+^ indicator bacteria tested. Figure 1 represents the number of G^+^ bacteria inhibited by each of the nine AP-bacteria, grouping the indicators in six categories: methicillin-resistant and -susceptible (MR and MS, respectively) *Staphylococcus, Enterococcus, M. luteus, L. monocytogenes*, and *S. suis.* Nevertheless, the AP staphylococci did not show AA against the two G– isolates tested (*E. coli* and *P. aeruginosa*). For this reason, all the next steps were performed only with G^+^ indicator bacteria.

**TABLE 1:** Staphylococcal isolates evaluated^a^ for antimicrobial activity by the *spot-on-lawn* method against 14 indicator bacteria and antimicrobial-producing (AP) isolates detected.

Species	N° of isolates^a^	Origin (n° of isolates)		AP isolates	
		**Nasal**	**Tracheal**	**N° of AP isolates**	**Stork sample ID code (origin)^b^**
*S. aureus*	46	22	24	1	436 (T)
*S. hyicus*	1	0	1	1	538 (T)
*S. sciuri*	138	54	84	2	507 (T), 433 (T)
*S. epidermidis*	16	3	13	1	506 (T)
*S. lentus*	14	5	9	0	–
*S. chromogenes*	11	7	4	1	481 (N)
*S. xylosus*	8	6	2	0	–
*S. hominis*	7	0	7	1	507 (T)
*S. saprophyticus*	6	1	5	0	–
*S. simulans*	7	6	1	2	480 (N), 481 (N)
*S. haemolyticus*	3	0	3	0	–
*S. capitis*	1	0	1	0	–
*S. arlettae*	1	1	0	0	–
Total	259	105	154	9	–

^a^Isolates were obtained from a previous study (Abdullahi et al., 2023).

^b^T, tracheal; N, nasal.

In this respect, the isolate with broader AA was *S. hominis* X3764, that inhibited all but one G^+^ indicator (*L. monocytogenes*). The most susceptible indicator category was MS-staphylococci (inhibited by six AP-isolates, 66.7%), followed by enterococci, *M. luteus* and *S. suis* (44.4%) and MR-staphylococci (33.3%). Interestingly, one out of the nine AP-isolates (*S. sciuri* X4000) revealed AA against *L. monocytogenes.* Moreover, most of the AP-isolates showed AA against more than one G^+^ indicator category tested. However, *S. epidermidis* X3815 isolate revealed a narrow inhibition profile, inhibiting only the MS-staphylococci indicators (Figure 1).

**FIGURE 2:**
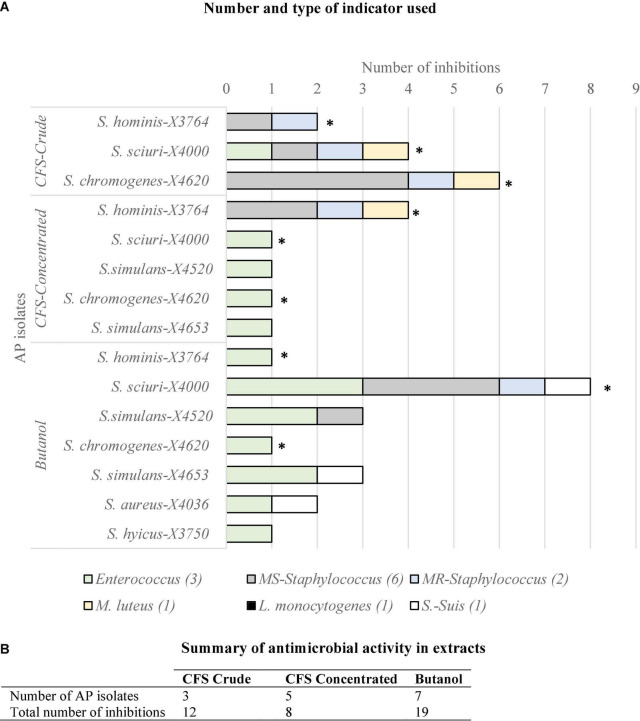
**(A)** Number of indicator bacteria (*n* = 14) inhibited by different preparations of the nine antimicrobial producer (AP) isolates of stork nasotracheal microbiota: crude CFS (boiled or filtrated), concentrated CFS, and after butanol extraction. **(B)** Number of AP isolates with antimicrobial activity in the three extract conditions tested and their respective total number of inhibitions. *Antimicrobial producing isolates with antimicrobial activity in the three conditions tested (*S. hominis* X3764, *S. sciuri* X4000, and *S. chromogenes* X4620).

Regarding extracts obtained in different conditions, only three AP-isolates (*S. hominis* X3764, *S. sciuri* X4000, and *S. chromogenes* X4620) revealed inhibitory capacity in all the conditions tested: crude CFS (filtered or boiled), CFS concentrated and butanol extraction. Moreover, other four isolates showed AA in the concentrated extracts: 55.6% revealed bioactivity in the concentrated CFS and this percentage increased to 77.8% after butanol treatment. Two additional isolates considered as AP by the *spot-on-lawn* method (*S. epidermidis* X3815 and *S. sciuri* X3763) were negative for all the extracts conditions against all the 14 G^+^ indicators tested (Figure 2).

Going deeper into the AA of the extracts obtained under different conditions, Figure 2 also represents the G^+^ indicator bacteria category inhibited in each of the extract conditions. This shows that extracts of all conditions tested (CFS crude and concentrated and butanol) inhibited MS-*Staphylococcus*, MR-*Staphylococcus*, and *Enterococcus* while none of them was active against *L. monocytogenes*. Interestingly, only the crude or concentrated CFS of some AP isolates showed AA against *M. luteus*, and *S. suis* was inhibited exclusively after butanol extraction of some AP isolates. Moreover, the three types of extracts were active against at least three indicator categories and as expected, concentrated CFS and butanol extracts showed a higher diversity of AP isolates and indicators.

### 3.2. Microbiota modulation

To analyse the ability for managing bacterial communities, the intra-sample AA of the nine AP isolates were firstly studied against the previously isolated G^+^ bacteria of the same sample as the producer isolate (Table 2). Only three of the AP-isolates (*S. hominis* X3764, *S. simulans* X4520, and *S. chromogenes* X4620) inhibited one of the G^+^ bacteria isolated in their respective samples (one *S. sciuri* and four *M. caseolyticus* isolates). Focusing on *S. hominis* X3764, it revealed AA against one of the two *S. isolates* isolates recovered in the same stork tracheal sample. However, *S. hominis* X3764 did not show antimicrobial activity against co-habitant *E. faecalis* isolates. In the same way, the AP isolates *S. simulans* X4520 and *S. chromogenes* X4620 inhibited the *M. caseolyticus* isolates co-habitant in the same nasotracheal sample when used as indicator bacteria (Table 2).

**TABLE 2:** Activity of the antimicrobial-producing (AP) isolates against Gram-positive (G^+^) bacteria cohabiting in the same stork (intra-activity) or representative of the nasotracheal microbiota of the storks (inter-activity).

Stork sample/origin	AP-isolate	Intra-activity^a^	Inter-activity^b^
538/Tracheal	X3750-S. *hyicus*	*S. sciuri, S. lentus,* *S. saprophyticus*	*S. aureus, E. durans, E. hirae, M. caseolyticus, M. luteus, Glutamicibacter sp., C. falsenii, C. aurimucosum, Corynebacterium sp.*
507/Tracheal	X3763-S. *sciuri*	*E. faecalis, S. sciuri*	None
507/Tracheal	X3764-S. *hominis*	***S. sciuri*,** *E. faecalis*	*S. sciuri, S. aureus, S. chromogenes, S. epidermidis, S. xylosus,* *S. lentus, S. saprophyticus, S. hyicus, S. arlettae, S. capitis,* *E. faecium, E. gallinarum, E. durans, E. hirae, M. caseolyticus,* *L. garvieae, M. luteus, V. lutrae, Glutamicibacter sp., Corynebacterium sp., C. falsenii, C. aurimucosum*
506/Tracheal	X3815-S. *epidermidis*	*Corynebacterium sp., E. faecium.*	*S. aureus, S. capitis*
433/Tracheal	X4000-S. *sciuri*	*E. faecalis, S. sciuri*	*E. durans, Glutamibacter sp.*
436/Tracheal	X4036-S. *aureus*	*S. sciuri, E. hirae, S. aureus, S. sciuri*	None
480/Nasal	X4520*-S. simulans*	***M. caseolyticus*,** *S. xylosus, S. sciuri*	*M. caseolyticus, E. durans, Glutamicibacter sp.* *C. falsenii, Corynebacterium sp., C. aurimucosum*
481/Nasal	X4620-*S. chromogenes*	***M. caseolyticus*,** *S. chromogenes*	*S. lentus, S. simulans*
481/Nasal	X4653-S. *simulans*	*M. caseolyticus,* *S. chromogenes*	*E. durans, M. caseolyticus, Glutamicibacter sp., C. falsenii*, *Corynebacterium sp., C. aurimucosum*

^a^For intra-activity, the G^+^ species cohabitant with AP-isolate in the same stork sample, that showed inhibition activity, are marked in bold.

^b^For inter-activity, 30 isolates of 29 different G^+^ species representative of the G^+^ nasotracheal diversity of storks were tested as indicator bacteria (see Table 3 and Supplementary Table 2).

In addition, the inter-sample AA of the nine AP isolates was tested against a collection of 30 G^+^ bacteria of 29 different species and nine genera representative of the G^+^ bacterial diversity of the stork nasotracheal microbiota (Table 3). The high AA of *S. hominis* X3764, inhibiting 73% of the isolates selected as a representative G^+^ microbial stork community is noteworthy. On the other hand, six AP isolates revealed AA against 7–33% of the G^+^ indicators tested. However, *S. sciuri* X3763 and *S. aureus* X4036 isolates lacked inhibitory capacity against any of those indicator bacteria (Table 3). Focusing on the AA of these AP-isolates, *Glutamicibacter sp*., (66.7%), *M. caseolyticus*, and *E. durans* (55.6%, respectively) were the most susceptible indicator bacteria inhibited by of AP-isolates (Table 3).

**TABLE 3:** Antimicrobial activity of the nine antimicrobial-producing (AP) isolates against a representative Gram-positive stork microbial community (30 isolates of 29 species used as indicator bacteria).

		Antimicrobial producing isolates									
**Species of indicator bacteria**	**Isolate ID code**	***S. hyicus* X3750**	***S. sciuri* X3763**	***S. hominis* X3764**	***S. epidermidis* X3815**	***S. sciuri* X4000**	***S. aureus* X4036**	***S. simulans* X4520**	***S. chromogenes* X4620**	***S. simulans* X4653**	**N° of AP isolates that inhibited the indicator bacteria (%)**
*S. sciuri*	X4121	–	–	+	–	–	–	–	–	–	1 (11%)
*S. aureus*	X4013	+	–	+	+	–	–	–	–	–	3 (33%)
*S. aureus*	X4409	+	–		–	–	–	–	–	–	1 (11%)
*S. chromogenes*	X4697	–	–	+	–	–	–	–	–	–	1 (11%)
*S. epidermidis*	X4146	–	–	+	–	–	–	–	–	–	1 (11%)
*S. xylosus*	X4413	–	–	+	–	–	–	–	–	–	1 (11%)
*S. lentus*	X4149	–	–	+	–	–	–	–	+	–	2 (22%)
*S. simulans*	X4525	–	–		–	–	–	–	+	–	1 (11%)
*S. hominis*	X3726	–	–		–	–	–	–	–	–	0
*S. saprophyticus*	X4145	–	–	+	–	–	–	–	–	–	1 (11%)
*S. hyicus*	X3750	–	–	+	–	–	–	–	–	–	1 (11%)
*S. haemolyticus*	X3784	–	–		–	–	–	–	–	–	0
*S. arlettae*	X4721	–	–	+	–	–	–	–	–	–	1 (11%)
*S. capitis*	X3968	–	–	+	+	–	–	–	–	–	2 (22%)
*S. pasteuri*	X4093	–	–		–	–	–	–	–	–	0
*E. faecalis*	X4126	–	–		–	–	–	–	–	–	0
*E. faecium*	X4688	–	–	+	–	–	–	–	–	–	1 (11%)
*E. gallinarum*	X4634	–	–	+	–	–	–	–	–	–	1 (11%)
*E. durans*	X4532	+	–	+	–	+	–	+	–	+	5 (56%)
*E. canis*	X3928	–	–		–	–	–	–	–	–	0
*E. hirae*	X4037	+	–	+	–	–	–	–	–	–	2 (22%)
*M. caseolyticus*	X4488	+	–	+	–	–	–	+	+	+	5 (56%)
*Lactococcus garvieae*	X4417	–	–	+	–	–	–	–	–	–	1 (11%)
*Streptococcus gallolyticus*	X4698	–	–		–	–	–	–	–	–	0
*M. luteus*	X4481	+	–	+	–	–	–	–	+	–	3 (33%)
*Vagococcus lutrae*	X4122	–	–	+	–	–	–	–	–	–	1 (11%)
*Glutamicibacter* sp.	X4102	+		+		+		+	+	+	6 (67%)
*C. falsenii*	X4270	+	–	+	–	–	–	+	–	+	4 (44%)
*Corynebacterium* sp.	X4486	+	–	+	–	–	–	+	–	+	4 (44%)
*C. aurimucosum*	X4660	+	–	+	–	–	–	+	–	+	4 (44%)
*n*=30 (%)*		10 (33%)	0	22 (73%)	2 (7%)	2 (7%)	0	6 (20%)	5 (17%)	6 (20%)	–

*Number of indicator bacteria (%) inhibited by each antimicrobial-producing (AP) isolate. Color: in dark green are marked the AP isolate with the strongest inhibition capacity against the major number of indicators and the indicator isolate that was inhibited by a higher number of AP isolates. In soft gray is shaded all the positive interactions of the AP isolates against the indicators.

However, considering each AP-isolate independently, the isolates of some species as *S. hominis, S. haemolyticus*, *S. pasteuri*, *E. faecalis, E. canis*, and *S. gallolyticus* were completely resistants to the antimicrobial activity of all the AP-isolates tested. Moreover, it is to note that some of those species (*E. faecalis*) were isolated in the same sample as the AP strains X3764 and X4000 (Table 3).

### 3.3. Characterization of the antimicrobial compound

It is of interest the detection of two AP-isolates (*S. hominis* X3764 and *S. sciuri* X4000) with the widest AA profile and strong inhibitory capacity in their respective extracts obtained in all conditions tested: crude (boiled and filtered), concentrated CFS and butanol extraction. These isolates were selected, and the susceptibility of their extracts to the enzymatic activity of trypsin, α-chymotrypsin, proteinase-K, papain, and protease was tested to verify the peptidic nature of the antimicrobial substances of AP isolates. The absence of AA against MRSA and MRSP indicators in the CFS of AP isolates after enzymatic treatment allowed to confirm the peptide nature of the antimicrobial substance present on these isolates.

Moreover, the presence of 22 bacteriocin structural genes were analysed by PCR and sequencing in the nine AP isolates. Genes encoding lantibiotic-like antimicrobial peptides were detected in all the nine AP isolates (Table 4).

**TABLE 4:** Characteristics of the nine antimicrobial producer isolates of the nasotracheal microbiota of storks.

Isolate	Stork sample ID code (origin)^a^	AMR phenotype^b^	Bacteriocin genes
*S. aureus* X4036	436 (T)	Susceptible	Lantibiotic-like
*S. hyicus* X3750	538 (T)	Susceptible	Lantibiotic-like
*S. sciuri* X3763	507 (T)	PEN	Lantibiotic-like
*S. sciuri* X4000	433 (T)	PEN, CIP	Lantibiotic-like
*S. epidermidis* X3815	506 (T)	ERY	Lantibiotic-like
*S. chromogenes* X4620	481 (N)	Susceptible	Lantibiotic-like
*S. hominis* X3764	507 (T)	PEN, FOX, ERY	Lantibiotic-like
*S. simulans* X4520	480 (N)	PEN	Lantibiotic-like
*S. simulans* X4653	481 (N)	Susceptible	Lantibiotic-like

^a^T, tracheal; N, nasal.

^b^Antibiotics tested were the following ones: PEN, penicillin; FOX, cefoxitin; ERY, erythromycin; CIP, ciprofloxacin; oxacillin, clindamycin, tetracycline, gentamicin, tobramycin, linezolid, chloramphenicol, trimethoprim-sulfamethoxazole, and mupirocin; AMR: antimicrobial resistance.

### 3.4. Antibiotic resistance profile of the AP isolates

Antibiotic susceptibility testing was performed with the collection of nine AP isolates. In this sense, 44.4% of the AP isolates showed susceptibility to the antimicrobial agents tested and the remaining isolates presented low resistance rates, highlighting the absence of MDR isolates. In this respect, 44.4% of the isolates showed resistance to penicillin, 22.2% to erythromycin and only 11.1% revealed resistance to cefoxitin or ciprofloxacin (Table 4).

## 4. Discussion

Antimicrobial resistance is a dynamic and multifaceted One-Health problem involving humans, animals, and the environment (Prestinaci et al., 2015). In this respect, the urgency to find new alternatives to antibiotics has induced the scientific community to focus on the antimicrobial substances produced by bacteria isolated from natural sources among which bacteriocins are of particular interest.

However, studies reporting the frequency of antimicrobial activity in bacteria of wildlife and livestock animals are limitated (Poeta et al., 2007; Almeida et al., 2011), and those focused on *Staphylococcus* are even scarcer (Fernández-Fernández et al., 2022), and storks have never been studied in this respect. The current work represents to the best of our knowledge, the largest study of antimicrobial activity, as well as the presence of bacteriocin structural genes in nasotracheal *Staphylococcus* isolates recovered from healthy wild storks.

Considering the inhibitory capacity of a collection of 259 *Staphylococcal* isolates recovered from nasotracheal samples of healthy storks in this study, nine of the isolates (3.5%) revealed antimicrobial activity against at least one of the 14 G^+^ indicators tested including methicillin-resistant and susceptible *Staphylococcus, Enterococcus*, and *L. monocytogenes*, among others. The AP isolates did not show antimicrobial activity against Gram-negative bacteria, such as *E. coli* and *P. aeruginosa*. Recent studies have detected *Staphylococcus* with antimicrobial activity among staphylococci of wild mammals and birds (excluding storks) (Fernández-Fernández et al., 2022), human nares (Janek et al., 2016), and those recovered from food (Van der Veken et al., 2020).

Among the *Staphylococcus* genus, several bacteriocins have been isolated from commensal CoNS species. Many of them display inhibitory activity against *S. aureus*, a CoPS widely considered an important pathogen of both humans and animals and implicated in a wide range of infections (Laux et al., 2019; Newstead et al., 2020).

To better characterize the inhibitory effect of the identified AP isolates, the antimicrobial activity of CFS was studied. However, bacteriocin production is costly and it is often regulated depending on cell density and environmental factors (Heilbronner et al., 2021), so we decided to extract the antimicrobial compounds from the inner cell. Thus, three of the nine AP isolates showed inhibition capacity in all the extract conditions tested (CFS crude or concentrated as well as butanol extract). However, when concentrating the extracts, most of the AP-isolates revealed bioactivity mainly against *Enterococcus* indicator bacteria. Nevertheless, two AP isolates (detected by the *spot-on-lawn* method) were negative for all the extract conditions and against all the indicators tested.

Regarding genetics, the nine AP isolates detected were positive for a lantibiotic-like structural gene although we can not discard that other structural bacteriocin genes, not tested in this study or not already described, could be responsible for the antimicrobial activity. A high variety of lantibiotics have been reported on *Staphylococcus* isolates such as epidermin, epilancins, and various nukacins, among others, showing antimicrobial activity exclusively against G^+^ bacteria (Laux et al., 2019).

According to microbiota composition, each community reflects specific niche conditions, and associations among species and concrete locations have been described (Wei et al., 2022). On the other hand, it is widely accepted that antibacterial molecules that inhibit major microbial competitors have a particularly important role in shaping the microbiome (Krismer et al., 2017; Lewis and Pamer, 2017; García-Bayona and Comstock, 2018).

Based on bacteriocin effects on the microbial community, the present study demonstrates the strong inhibitory capacity of the recovered AP-isolates. Especially, *S. hominis* X3764 could act as a microbiota modulator due to its high inter-sample AA against 73% of the G^+^ representative stork community and the intra-sample activity, inhibiting the *S. sciuri* isolate recovered from stork 507. In this respect, potent antimicrobial activity has been previously reported in nasal staphylococci of human origin (80%) against bacteria of the nasal ecosystem (Janek et al., 2016).

As for the nasal microbiome, *S. aureus* is also regarded as a human commensal that colonizes asymptomatically about 30% of human nares (Laux et al., 2019). However, nasal carriage of *S. aureus* predisposes to invasive infection (Zipperer et al., 2016). For example, it has been described that nasal *S. lugdunensis* can prevent *S. aureus* colonization by producing an unusual antimicrobial compound termed lugdunin (Zipperer et al., 2016). Moreover, *S. epidermidis* has been reported as an *S. aureus* inhibitor although there is no clear correlation between the absence of *S. aureus* with the presence of *S. epidermidis* (Bierbaum and Sahl, 2009; Iwase et al., 2010; Yan et al., 2013).

On the other hand, recent studies with antimicrobial-producing isolates indicate that a specific bacteriocin may not affect all microbiome members equally and may only affect those with closer physical proximity. Therefore, bacteriocin-producing isolates have an important role in niche competition and colonization conferring to the producer an ecological superiority against other microbiome constituents without natural bioactive compounds that allows maintaining of stable communities and can lead to the redistribution of microbiome members into sub-niches (Heilbronner et al., 2021).

Although migratory and resident wild birds are not implicated directly in the development of antimicrobial resistance, they are considered important reservoirs and vectors of zoonotic and antimicrobial-resistant bacteria (Gómez et al., 2016; Wang et al., 2017; Abdullahi et al., 2021). In combating the global antimicrobial resistance problem, they could be be considered due to their potential ability to carry *Staphylococcus* spp. which produce effective antimicrobial compounds of relevance in biomedical and food production sciences. Fortunately, half of the AP isolates were susceptible to all antibiotics tested and none of them was MDR.

## 5. Conclusion

Exploring the mechanisms of how bacteriocins affect microbiota dynamics requires an improved understanding of the functions of these diverse molecules. Many studies have been carried out to elucidate the exclusion mechanisms of *S. aureus* from the human nose. However, more studies should be undertaken to clarify which antimicrobial substances are produced by nasal commensals among other strategies used in their competition with nasal microbiota for nutrients and adhesion sites.

In this respect, the two highly AP isolates identified in this study (*S. hominis* X3764 and *S. sciuri* X4000) could be excellent candidates for further studies as an alternative to the alarming situation of antibiotic resistance. This highlights the relevant role of the nasotracheal microbiota of storks as a model for the control of bacterial communities by bacteriocin-producing isolates and their transmission to humans, other animals, and the environment.

Moreover, the evaluation of bacteriocins production by staphylococci from wild animal can contribute to their potential application on other hosts and ecosystems.

## Data availability statement

The original data presented in this study are included in the article/Supplementary material, further inquiries can be directed to the corresponding author.

## Ethics statement

The sampling procedures performed in the previous study in which the staphylococci isolates were obtained (Abdullahi et al., 2023, in press), and were approved by the ethical committee for animal experimentation of the University of Castilla-La Mancha and authorized by the regional government of Castilla-La Mancha (permit no. VS/MLCE/avp_21_198); moreover, all applicable international, national, and/or institutional guidelines for the care and ethical use of animals, specifically directive 2010/63/EU and Spanish laws 9/2003 and 32/2007, and RD 178/2004 and RD 1201/2005 were followed.

## Author contributions

CT, CL, and RF-F designed the study, made the first analysis of the data, and prepared the draft of the manuscript. RF-F, UH, INA, AU, and CG-A performed sampling, recovery of isolates, and experimental work. RF-F, SG-V, and AM participated in the graphical design of data and some methodological issues. CT and CL supervised the study. All authors read and agreed to the published version of the manuscript.
