# Supplementary material for: Detection of antimicrobial producing Staphylococcus from migratory birds: Potential role in nasotracheal microbiota modulation
Source: Front Microbiol. 2023 Apr 11;14:1144975. doi: 10.3389/fmicb.2023.1144975 (PMC10126283; doi:10.3389/fmicb.2023.1144975)
Supplement: Supplementary file 1 [file Data_Sheet_1.docx]

**Supplementary material**

**Table S1**. Characteristics of the 14 Gram-positive (G^+^) indicator bacteria used in this study for the screening of antimicrobial activity production in the 259 staphylococci of storks and the two Gram-negative indicator bacteria tested for the 9 antimicrobial-producing (AP) isolates.

| **Specie (nº of isolates)** | **Isolate ID code** | **Origin** | **Relevant Antimicrobial Resistance Phenotype/Genotype** | **Reference** |
| --- | --- | --- | --- | --- |
| *Staphylococcus aureus* (2) | C1570 | Human-skin | Methicillin/*mec*A (MDR) | UR collection |
|  | ATCC29213 | Reference strain |  | ATCC collection |
| *Staphylococcus pseudintermedius* (2) | C2381 | Pet-nasal | Methicillin/*mec*A | UR collection |
|  | C3468 | Pet-nasal |  | UR collection |
| *Staphylococcus lugdunensis* (1) | C10107 | Human-blood |  | UR collection |
| *Staphylococcus epidermidis* (1) | C2663 | Human-blood | Methicillin/*mec*A and Linezolid | UR collection |
| *Staphylococcus sciuri* (1) | C9780 | Wild boar-nasal |  | UR collection |
| *Staphylococcus delphini* (1) | C9459 | Vulture-nasal |  | UR collection |
| *Enterococcus faecalis* (1) | ATCC29212 | Reference strain |  | ATCC collection |
| *Enterococcus faecium* (1) | C2321 | Human-wound | Vancomycin*/van*A | UR collection |
| *Enterococcus cecorum* (1) | X3809 | Storks-nasal |  | UR collection |
| *Listeria monocytogenes* (1) | CECT4032 | Reference strain |  | CECT collection |
| *Micrococcus luteus* (1) | CECT241 | Reference strain |  | CECT collection |
| *Streptococcus suis* (1) | X2060 | Pig |  | UR collection |
| *Escherichia coli* (1) | ATCC25922 | Reference strain |  | ATCC Collection |
| *Pseudomonas aeruginosa* (1) | PAO1 | Reference strain |  |  |

**Table S2.** Gram-positive bacteria (30 isolates of 29 different species and 9 genera) selected as representative of the nasotracheal diversity of storks used as indicator bacteria in inter-sample antimicrobial production assays (strains selected of Abdullahi et al., 2023, in press)

| **Species of indicator bacteria** | **Isolate ID code** |
| --- | --- |
| *Staphylococcus sciuri* | X4121 |
| *S. aureus* | X4013 |
| *S. aureus* | X4409 |
| *S. chromogenes* | X4697 |
| *S. epidermidis* | X4146 |
| *S. xylosus* | X4413 |
| *S. lentus* | X4149 |
| *S. simulans* | X4525 |
| *S. hominis* | X3726 |
| *S. saprophyticus* | X4145 |
| *S. hyicus* | X3750 |
| *S. haemolyticus* | X3784 |
| *S. arlettae* | X4721 |
| *S. capitis* | X3968 |
| *S. pasteuri* | X4093 |
| *Enterococcus faecalis* | X4126 |
| *E. faecium* | X4688 |
| *E. gallinarum* | X4634 |
| *E. durans* | X4532 |
| *E. canis* | X3928 |
| *E. hirae* | X4037 |
| *Macrococcus caseolyticus* | X4488 |
| *Lactococcus garvieae* | X4417 |
| *Streptococcus gallolyticus* | X4698 |
| *Micrococcus luteus* | X4481 |
| *Vagococcus lutrae* | X4122 |
| *Glutamicibacter* sp.* | X4102 |
| *Corynebacterium* sp*.* | X4486 |
| *C. falsenii* | X4270 |
| *C. aurimucosum* | X4660 |

**Glutamicibacter* has been recently recognised into a new genus and it was considered previously as *Arthrobacter*.
